# Biological Processes Underlying Co-Use of Alcohol and Nicotine: Neuronal Mechanisms, Crosstolerance, and Genetic Factors

**Published:** 2006

**Authors:** Douglas Funk, Peter W. Marinelli, Anh D. Lê

**Affiliations:** Douglas Funk, Ph.D., is a staff scientist and Peter W. Marinelli, Ph.D., is a postdoctoral fellow at the Centre for Addiction and Mental Health in Toronto, Canada. Anh D. Lê, Ph.D., is a senior scientist at the Centre for Addiction and Mental Health and an associate professor in the Departments of Pharmacology and Psychiatry at the University of Toronto, Canada

**Keywords:** Alcohol and tobacco, alcohol and other drug (AOD) use, abuse, and dependence, nicotine dependence, dual addiction, brain, ventral tegmental area, mesolimbic dopamine system, animal models, genetic theory of alcohol and other drug use (AODU), cross-tolerance

## Abstract

Alcohol and nicotine are two of the oldest and most commonly used recreational drugs, and many people use both of them together. Although their ready availability likely contributes to the strong correlation between alcohol and nicotine use, several lines of evidence suggest that biological factors play a role as well. For example, both alcohol and nicotine act on a brain system called the mesolimbic dopamine system, which mediates the rewarding and reinforcing properties of both drugs. Modification of the activities of the mesolimbic dopamine system can interfere with the effects of both alcohol and nicotine. Another mechanism that may contribute to alcohol–nicotine interactions is cross-tolerance to the effects of both drugs. Finally, genetic studies in humans and of selectively bred mouse and rat strains suggest that shared genetic factors help determine a person’s liability to use or abuse both alcohol and nicotine.

Alcohol and nicotine are two of the most commonly used drugs in the world, and many people use both of them, often together. In humans, alcohol and nicotine use are highly correlated. For example, the prevalence of smoking is about three times higher in alcoholics than in the general population (Substance Abuse and Mental Health Services Administration 2005). This relationship between alcohol and nicotine consumption is especially interesting because the two drugs are rather dissimilar in their mechanisms of action and their effects:

- Nicotine acts on the brain by directly binding to and activating a molecule called the nicotinic acetylcholine receptor, which is found on certain brain cells. In contrast, alcohol does not bind directly to any one receptor type.
- Alcohol usually is classified as a depressant, impairs alertness, and can have anticonvulsant effects. Conversely, nicotine primarily has stimulant effects, increases alertness, and can trigger convulsions.
- The withdrawal symptoms induced by the two drugs following chronic administration differ considerably.

The fact that alcohol and tobacco are readily and legally available likely contributes to their co-use. Over the past two decades, however, it has become increasingly clear that biological factors also contribute to the concurrent use of alcohol and nicotine—that is, certain common physiological mechanisms may account for people’s liability to use both alcohol and nicotine. Several such mechanisms have been suggested. For example, both alcohol and nicotine may enhance a person’s motivation to also consume the other drug by acting on a common target in the brain that is responsible for the reinforcing effects of both drugs. Alternatively, cross-tolerance between the two drugs may reduce the drugs’ aversive effects and motivate people to use more of the drugs in order to achieve the same rewarding effects. (The concept of cross-tolerance will be explained in more detail in the section “Tolerance and Cross-Tolerance.”) The study of this possible mechanism, however, is complicated by the fact that both alcohol and nicotine can exert different effects on a variety of behavioral and physiological variables, depending on the amount of the drugs consumed (Moore et al. 1993; Nanri et al. 1998; Schaefer and Michael 1986). Finally, several recent studies have suggested that a shared genetic component may predispose people to the use or abuse of both alcohol and nicotine.

This article reviews and evaluates the evidence for these three biological mechanisms that potentially contribute to the co-use of alcohol and nicotine. After reviewing human and animal studies that demonstrate how alcohol and nicotine interact in producing their rewarding effects, the article presents the evidence that a pathway in the brain—the mesolimbic dopamine system—participates in this interaction. The possibility that tolerance and cross-tolerance to the effects of alcohol and nicotine contribute to their co-use will be discussed, as will the evidence for a shared genetic predisposition to concurrently use or abuse alcohol and nicotine. Additional important lines of evidence supporting the existence of an interaction between alcohol and nicotine, such as variability in the receptors that are affected by alcohol and nicotine use (Connor et al., in press) or alcohol’s effects on nicotinic receptor function (Bowers et al. 2005; Butt et al. 2003; Tritto et al. 2001), are reviewed elsewhere in this issue.

## Interaction Between the Reinforcing Effects of Nicotine and Alcohol

The interactions between the reinforcing effects of nicotine and alcohol have been studied both in humans and in animal models.

### Human Studies

Although it has long been assumed that smoking enhances the pleasurable effects of drinking, this only recently has been tested experimentally. In one study, investigators asked male smokers to smoke regular or denicotinized cigarettes while performing a progressively more demanding task that was rewarded with the opportunity to drink alcoholic beverages. Under these conditions, participants who smoked nicotine- containing cigarettes worked harder and drank more alcohol than did those who smoked the denicotinized cigarettes (Barrett et al. 2006). Other investigators have demonstrated the converse relationship—that is, that alcohol can enhance the self-reported pleasure derived from cigarette smoking (Rose et al. 2004).

Another approach to analyzing alcohol–nicotine interactions involves a substance called mecamylamine, a nicotinic receptor antagonist. The term “antagonist” means that this agent binds to nicotinic receptors found on brain cells, thereby preventing nicotine from acting on these receptors. Chi and de Wit (2003) found that social drinkers who were treated with mecamylamine experienced lesser euphoric and stimulant-like effects after consuming alcohol than they normally did. This observation strongly suggests that alcohol must interact in some way with nicotinic receptors to induce its pleasurable effects. Additional studies have shown that the interaction between alcohol and nicotine is complex and is influenced by numerous modulating factors, such as the person’s age and gender (Acheson et al. 2006; Grant et al. 2006).

### Animal Studies

For the most part, the data obtained using animal models agree with the findings of human studies. An early study assessed the effects of nicotine on alcohol consumption in alcohol-drinking rats that were surgically implanted with nicotine-releasing capsules under the skin, similar to the present-day “nicotine patch.” The researchers found that compared with control animals, rats implanted with these capsules dramatically increased their alcohol consumption over several days (Potthoff et al. 1983).

Since then, other researchers have attempted to replicate these findings by using either repeated daily injections or subcutaneous capsules to chronically deliver nicotine. In general, these studies found that nicotine increased alcohol consumption when the animals had access to an alcohol-containing drinking bottle (Larsson and Engel 2004; Smith et al. 1999) or when they had to work (i.e., press a lever) to obtain alcohol (Clark et al. 2001; Lê et al. 2000). Consistent with these findings, and with those of the human studies described above, treatment with mecamylamine to block nicotinic receptors resulted in reduced alcohol consumption in rats (Larsson and Engel 2004; Lê et al. 2000).

Another animal model that has provided further evidence for nicotine-induced enhancement of the motivation to obtain alcohol is the relapse or reinstatement procedure. In this model, animals are first trained to press a lever in order to obtain alcohol. Once stable lever pressing has been established, the behavior is extinguished by withholding the alcohol—that is, lever presses no longer result in alcohol delivery. Under these conditions, the animals eventually will cease to press the lever. Using this design, Lê and colleagues (2003) demonstrated that nicotine injections could reinstate lever pressing for alcohol in a dose-dependent manner. This suggests that nicotine can affect brain pathways that control alcohol-seeking behavior.

Taken together, these studies provide strong evidence that alcohol and nicotine can potentiate each other’s rewarding effects in humans and laboratory animals. The following sections describe potential mechanisms that underlie this relationship.

## Potential Mechanisms Underlying Alcohol–Nicotine Interactions

### Neural Mechanisms

**Figure 1:**
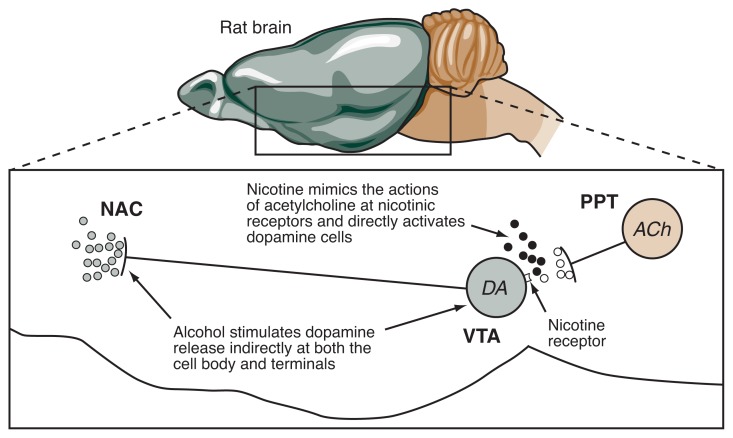
Interactive effects of alcohol and nicotine on dopamine release in the rat brain. Under normal conditions, neurons from the pedunculopontine tegmental nucleus (PPT) that release the neurotransmitter acetylcholine (ACh) extend to the ventral tegmental area (VTA). Released ACh (open circles) stimulate nicotinic receptors on neurons that in turn release the neurotransmitter dopamine (DA) in several brain regions, including the nucleus accumbens (NAC). When nicotine (black circles) enters the brain from the circulation, it acts on the nicotinic receptors on the dopamine-containing neurons in the VTA, resulting in increased dopamine release (shaded circles) in the NAC. Alcohol also causes dopamine release in the VTA and NAC through an unknown, but likely indirect, mechanism. The combined effects of alcohol and nicotine can enhance dopamine release in the NAC.

Much of the work exploring the brain structures and mechanisms that underlie the rewarding effects of alcohol and nicotine (and their interactions) has focused on a brain pathway known as the mesolimbic dopamine system (see Figure 1). This pathway originates in a cluster of nerve cells (neurons) in a region of the midbrain called the ventral tegmental area (VTA). These cells, which release the chemical messenger (i.e., neurotransmitter) dopamine to transmit signals to other brain cells, project to and interact with various brain structures involved in reward, emotion, memory, and cognition. Of these brain structures, an area in the forebrain known as the nucleus accumbens has been extensively studied with respect to its involvement in the reinforcing effects of drugs, including alcohol and nicotine (Larsson and Engel 2004).

Many studies have established a role for the mesolimbic dopamine system in the motivation to seek alcohol or nicotine. The dopamine-releasing neurons of the VTA possess nicotinic receptors. Normally, these neurons receive signals from neurons in a brain region called the pedunculopontine tegmental nucleus (PPT) (Kitai et al. 1999). The PPT cells release the neurotransmitter acetylcholine from their projections to the VTA, which then acts on the nicotinic receptors there, thereby stimulating the VTA cells to release dopamine in its various target brain regions.

Some evidence suggests that nicotine exerts its reinforcing effects by stimulating the nicotinic receptors found on VTA neurons. For example, researchers found that injections into the VTA of agents that block nicotinic receptors can reduce nicotine self-administration (Corrigall et al. 1994). This observation indicates that in order to exert its reinforcing effects, nicotine needs to interact with these receptors in the VTA.

Several studies have examined the role of the mesolimbic dopamine system in alcohol self-administration, for example, by evaluating the effects of agents that either stimulate or prevent the release of dopamine from the VTA cells. These studies have produced variable results, especially if these agents were injected systemically (e.g., into the blood stream) and therefore were distributed through all tissues of the organism (Gonzales et al. 2004). More consistent results were obtained from studies in which the agents were injected into specific brain regions. For example, directly injecting dopamine-releasing agents into the nucleus accumbens can increase alcohol drinking. Conversely, injection into the VTA of agents that reduce dopamine release in the nucleus accumbens decreases drinking (Gonzales et al. 2004).

Only a few studies have specifically examined the role of the mesolimbic dopamine system in the interactions between alcohol and nicotine. One such study demonstrated that pharmacological blockade of nicotinic receptors in the VTA suppresses alcohol intake, further supporting the hypothesis that the rewarding effects of alcohol depend in some way on nicotinic receptors (Soderpalm et al. 2000).

Other studies have used a technique known as *in vivo* microdialysis, which enables researchers to measure the release of neurotransmitters in specific brain regions of freely behaving animals. The results of these studies also support the assumption that alcohol and nicotine produce synergistic behavioral effects by acting on the mesolimbic dopamine system. For example, in investigations using *in vivo* microdialysis, both alcohol and nicotine stimulated the release of dopamine in the nucleus accumbens (Di Chiara and Imperato 1988). Similarly, direct injection of nicotine into the VTA stimulated dopamine release in the nucleus accumbens (Tizabi et al. 2002). Interestingly, this effect can be further enhanced by combining nicotine injections into the VTA with systemic alcohol injections, suggesting a mechanism for the alcohol–nicotine interactions observed in the self-administration studies described above.

Finally, the role of the mesolimbic dopamine system in mediating alcohol–nicotine interactions is supported even more strongly by the observation that blocking nicotine receptors in the VTA abolishes the alcohol-induced increase in dopamine release (Ericson et al. 2003; Tizabi et al. 2002).

Taken together, all these data indicate that the interaction of nicotine and alcohol depends on the activity of the mesolimbic dopamine pathway. It is likely, however, that other mechanisms, which have yet to be fully investigated, also influence the alcohol–nicotine interactions. For example, the brain’s response to stress is thought to be intimately related to the reinforcing properties of drugs of abuse (Piazza et al. 1996). Because both alcohol and nicotine have the potential to reduce general anxiety under specific conditions and to attenuate withdrawal symptoms, it may not be surprising that high levels of stress enhance the urge to consume alcohol and/or nicotine (Onaivi et al. 1989). On the other hand, acute alcohol or nicotine administration increases the level of stress hormones that circulate in the blood in a dose-dependent manner (Mendelson et al. 2005; Ogilvie and Rivier 1996). It therefore is possible that alcohol and nicotine act together on the stress-responsive pathways in the brain in a manner that promotes the concurrent use and abuse of both drugs.

### Tolerance and Cross-Tolerance

Tolerance is defined as a reduction in the response to a drug after repeated exposure to the drug. Development of tolerance to alcohol and other drugs is thought to be an important factor in the escalation of drug intake and the development of drug dependence. Both in humans and in animal models, repeated use of alcohol or nicotine results in tolerance to a variety of the drugs’ pharmacological effects (Perkins 2002; Suwaki et al. 2001).

The development of tolerance resulting from chronic exposure to one drug also can confer cross-tolerance to one or more other drugs. Although it is difficult to evaluate the development of cross-tolerance between alcohol and nicotine in humans because both drugs are so commonly used and abused together, cross-tolerance between alcohol and nicotine is well documented in animal models. For example, both alcohol and nicotine can lead to a lowering of the body temperature (i.e., have a hypothermic effect) and to a slowing of the heart rate (i.e., have a bradycardiac effect). Early studies in mice have demonstrated that chronic alcohol administration via a liquid diet, which induced tolerance to several behavioral effects of alcohol, also conferred cross-tolerance to the hypothermic and bradycardiac effects of nicotine (de Fiebre and Collins 1993). Conversely, nicotine-tolerant mice displayed cross-tolerance to the hypothermic and bradycardiac effects of alcohol.

The development of cross-tolerance between alcohol and nicotine may contribute to the concurrent use of both drugs in humans. One model explaining the reduced sensitivity (i.e., tolerance) to alcohol in smokers compared with nonsmokers posits a role for certain genes that might predispose individuals to both alcohol and nicotine use (Madden et al. 2000). (For more information on this model and the evidence supporting it, see the next section.) However, reduced sensitivity to the intoxicating effects of alcohol and nicotine resulting from repeated exposure to one drug alone might facilitate use of the other drug and account for co-abuse. For example, reduced sensitivity to alcohol may be an important determinant in the development of alcohol abuse and dependence (Schuckit and Smith 2001). Similarly, it is possible that reduced sensitivity to alcohol resulting from chronic exposure to nicotine might facilitate alcohol intake, and vice versa. Such a relationship could have important implications given the high prevalence of tobacco use initiation during adolescence. A plausible scenario is that after adolescent nicotine exposure, cross-tolerance to alcohol might facilitate later alcohol use.

## Genetic Factors

It has been known for many years that genetic factors contribute to the likelihood that a person will smoke or will become dependent on alcohol. More recently, the possibility that genetic factors may determine the co-use of these two drugs has received much attention (Tyndale 2003). Using a variety of genetic techniques, several studies have addressed this issue, and their results suggest a strong genetic influence on the liability to co-use alcohol and nicotine.

Studies of twins have been useful in uncovering the relative genetic and environmental contributions to the development of various diseases, including drug use disorders. Such studies have shown that compared with fraternal (i.e. dizygotic) twins who share about 50 percent of their genes (like other siblings), identical (i.e., monozygotic) twins who share 100 percent of their genes are twice as likely to be alcohol dependent or nicotine dependent if one of the pair is dependent. These findings indicate that more than 50 percent of a person’s liability to develop alcohol or nicotine dependence results from genetic influences (Carmelli et al. 1993; Enoch and Goldman 2001; Swan et al. 1997). A number of other studies using other genetic approaches support the idea that genetic factors account for the concurrent abuse of nicotine and alcohol (Bierut et al. 2004; Goldman et al. 2005; Swan et al. 1997; True et al. 1999).

These and other studies have led to the hypothesis that genetic factors mediate the concurrent abuse of nicotine and alcohol (Tyndale 2003). However, the findings of these human genetic studies can be criticized on the basis that combined expression of such traits is common in many psychiatric disorders (Goldman et al. 2005; Krishnan 2005). In addition, it is possible that alcohol use leads to nicotine use, or vice versa (Tyndale 2003). Finally, both alcohol and nicotine are readily available and, compared with illicit drugs, can be legally purchased in virtually any quantity by adults. All of these factors may contribute to the increased co-abuse of alcohol and nicotine and may therefore lead to a possible overestimation of a shared genetic component.

Another line of research that provides evidence for a genetic linkage between the effects of alcohol and nicotine involves the selective breeding of rats or mice for different responses to alcohol. These types of experiments have the advantage of being free of the confounding factors associated with human studies because the animals had never been exposed to drugs (i.e., are drug naïve) prior to being studied. Researchers have generated many strains of rats and mice that differ with respect to specific responses to alcohol or nicotine (de Fiebre et al. 2002; Tritto et al. 2001; Turek et al. 2005). Studies using such animal strains have demonstrated a substantial overlap between the sensitivity to alcohol and the sensitivity to nicotine. For example, mice or rat lines bred for differences in the sedative effects of alcohol also demonstrate differences in body temperature and locomotor responses to nicotine injections (de Fiebre et al. 1990).

**Figure 2:**
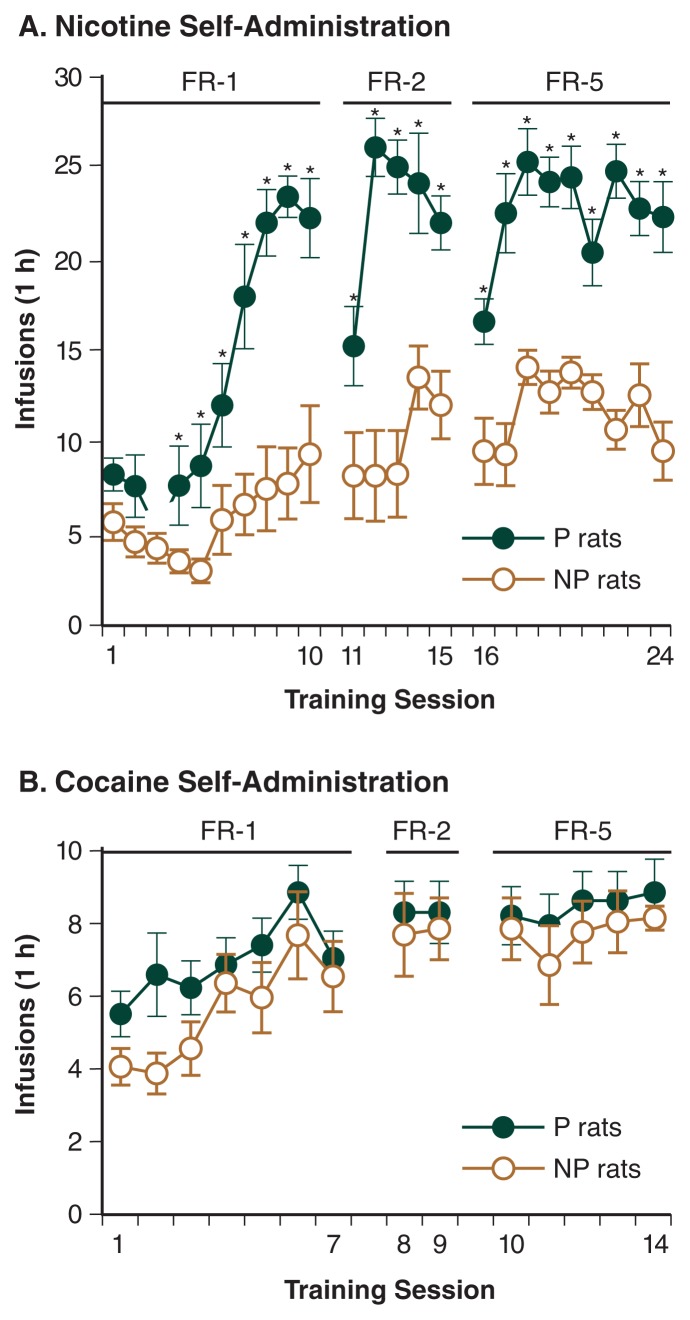
**A** P rats (closed symbols), which are genetically selected for high alcohol drinking, self-administer more nicotine intravenously than do NP rats (open symbols), which are selected for low drinking. The animals were allowed to self-administer nicotine during the daily 1-hour sessions under different reinforcement schedules^†^. The number of nicotine infusions earned by the animals during the first 24 days of nicotine self-administration (0.03 mg/kg per infusion) is shown. **B** The two strains do not differ in the amount of cocaine they self-administer intravenously. The number of cocaine infusions during the first 14 days of cocaine self-administration (0.75 mg/kg per infusion) is shown. SOURCE: Modified from Le et al., 2006. © 2006, Society for Neuroscience. ^*^Days when P rats self-administered significantly more nicotine that NP rats. ^†^With the FR-1 schedule, animals had to press a lever once to receive the infusion, with the FR-2 schedule they had to press the lever twice, and with the FR-5 schedule they had to press the lever five times.

Lê and colleagues (2006) recently conducted a series of experiments to test the hypothesis that shared genetic factors mediate vulnerability to both nicotine and alcohol. The investigators examined the nicotine-taking behavior of rats that were genetically selected for high alcohol intake (P rats) or low alcohol intake (NP rats).^1^ Alcohol-naïve animals from both of these strains were trained to press a lever to receive intravenous nicotine injections. The researchers found that the P rats self-administered greater amounts of nicotine than did the NP rats (Figure 2A). Moreover, if the lever-pressing behavior was extinguished by withholding the nicotine injections, the P rats demonstrated greater relapse to nicotine if they received a priming nicotine injection or if they were exposed to cues that had previously been associated with nicotine injection. These results suggest that P rats, which were selectively bred for high alcohol intake, find nicotine more rewarding than do NP rats, selectively bred for low alcohol intake.

Additional analyses of these animals demonstrated that the increased nicotine self-administration in P rats compared with NP rats was not attributable to learning deficits, because both P and NP rats rapidly learned to self-administer oral sucrose, a natural reinforcer. Nor were the differences attributed to a failure of NP rats to acquire nicotine self-administration, because these animals increased their lever pressing over several days and then maintained stable daily nicotine intake (Lê et al. 2006). Finally, the experiments showed that differences in self-administration were specific to nicotine, because P and NP rats self-administered equivalent amounts of the psychostimulant cocaine (Figure 2B). This disproves a possible alternative explanation for the P–NP differences, namely that P rats may be more sensitive to the rewarding effects of drugs in general and not only to the effects of alcohol and nicotine.

Taken together, these results obtained by Lê and colleagues (2006) suggest that nicotine has greater reinforcing or rewarding effects in animals that were genetically selected for high alcohol intake than in animals selected for low intake. These differences are genetically based and free of any confounding effects of prior alcohol exposure because the animals studied were alcohol naive. The findings of this study therefore provide further evidence that a shared genetic basis may underlie, at least in part, the tendency to use both alcohol and nicotine.

## Conclusions

Several lines of research suggest that there is a biological basis for the concurrent use or abuse of alcohol and nicotine. Although the specific mechanisms underlying the interaction of alcohol and nicotine are not yet fully known, there is evidence that the two drugs may act on the same brain pathways— particularly the mesolimbic dopamine system—to exert their rewarding effects. Cross-tolerance between alcohol and nicotine could also be a mechanism through which either drug enhances the reinforcing properties of the other. To date, the strongest evidence for a biological basis for alcohol and nicotine co-use may come from genetic studies. Both studies of human twins and studies conducted with selected strains of laboratory animals suggest that the predisposition to use alcohol and nicotine has a strong genetic component.

These findings may have important implications for the treatment of alcohol or nicotine dependence. Thus, selection of the most appropriate treatment approach may depend on whether or not the patient is co-dependent on both drugs. Future studies should be directed to refining our knowledge of the behavioral mechanisms and underlying neural pathways mediating the co-use of alcohol and nicotine in order to guide the development of more effective prevention and treatment strategies.

## Footnotes

1 These strains, which were bred at the University of Indiana, have been used for many years as a genetic animal model of alcoholism (Murphy et al. 2002). Although both strains were derived from the same parent stock, P rats consume an average of 5.7 g/kg alcohol per day, whereas NP rats barely drink 0.5 g/kg per day (Lumeng et al. 1995).
